# Stress and influencing factors of public health and preventive medicine students in the post-pandemic period: a cross-sectional study in Changsha, China

**DOI:** 10.3389/fpubh.2023.1227441

**Published:** 2023-08-01

**Authors:** Xiaofen Wang, Jiahui Zhang, Na Yang, Miliang Zou, Pingping He

**Affiliations:** ^1^Department of Nursing, School of Medicine, Hunan Normal University, Changsha, China; ^2^Department of Oncology, The Second Xiangya Hospital of Central South University, Changsha, China

**Keywords:** post-COVID-19 era, public health, preventive medicine, stress, influence factor

## Abstract

**Objectives:**

Little is known about the stress levels and associated factors of public health and preventive medicine students in the post-pandemic period. This study aims to investigate the stress levels of these students in the post-COVID-19 era and to determine the association of personal background, employment attitude, and psychological state with stress.

**Methods:**

A cross-sectional survey was conducted in March 2023 among 620 public health and preventive medicine students from two universities in Changsha, China. The survey included demographic characteristics, employment attitudes, perceived stress scale 10, general anxiety disorder 7, the University of California at Los Angeles loneliness scale 20, and the PTSD checklist-civilian version. Two-sided *t*-tests and ANOVA tests were used to compare the differences in PSS scores among variables, and multiple hierarchical regression analysis was used to evaluate the associated factors with stress.

**Results:**

The survey was completed by 504 students (mean age: 21.5 ± 2.6 years, 69.2% female). 24.8% of the students were screened for a high level of stress. 69.0% thought the epidemic positively impacted employment while 18.5% believed it had a negative impact. The results of regression analysis showed that older age (*B* = 0.42, *p* = 0.001), higher grade ( $B_{1}=$ 3.59, *p* < 0.001, $B_{2}=$ 4.57, *p* < 0.001), having internship experiences (*B* = 1.16, *p* = 0.006), having anti-epidemic experiences (*B* = 1.77, *p* < 0.001), believing that COVID-19 has a negative impact on employment (*B* = 2.56, *p* < 0.001), and having higher GAD scores (*B* = 0.64, *p* < 0.001) and UCLA scores (*B* = 0.07, *p* = 0.002) were significantly associated with high-stress levels. Conversely, being female (*B* = −1.64, *p* < 0.001) and believing that the pandemic had a positive impact on employment (*B* = −1.98, *p* = 0.001) were associated with low-stress levels.

**Conclusion:**

Public health and preventive medicine students in Changsha, China, experienced a high-stress level in the post-pandemic period, which was influenced by age, gender, grade, employment attitude, internship experience, anxiety, and loneliness. As one of the main guardians of the epidemic, these students should be given more attention and psychological interventions in the future.

## 1. Introduction

Since the coronavirus disease 2019 (COVID-19) was formally declared a global pandemic by the World Health Organization (WHO) in February 2020 (1), people all over the world have been fighting it for more than 3 years, but it has not completely left the human arena, and some countries are experiencing multiple waves of raised infections (2). As of March 2023, COVID-19 had caused more than 760 million confirmed cases and more than 6.9 million deaths worldwide (3), and the numbers continue to increase. In addition to posing a great threat to the physical health of individuals, the pandemic has also caused a series of psychological problems such as anxiety in the global population, affecting mental health outcomes, and even leading to cases of stereotyping and discrimination (4–6).

College students have inevitably developed various psychological problems during the pandemic. Since the pandemic has changed the original curriculum and increased concerns about future employment, medical students, as the main body of future clinical works, have been paid wide attention (7, 8). Even in the late stage of the pandemic, their psychological problems are still widely reported (9). A study involving 418 medical students showed that 93.2% of the students experienced anxiety, depression, and loneliness during the pandemic (10). An Australian study confirmed that 68% of medical students reported that their mental health deteriorated after the outbreak due to concerns about social relationships, study, and employment (11). Studies from China showed that the epidemic had affected the employment attitudes of medical students, students in hard-hit or high-incidence areas were less willing to choose epidemic-related majors (12), and 3.4% of college students reported symptoms of post-traumatic stress disorder in the post-pandemic era (9). However, these studies did not divide medical students in a more detailed way, and most of them focused on clinical medicine and nursing students, while studies on public health and preventive medicine students were scarcely reported.

In fact, public health and preventive medicine students have been hit hard by the pandemic, but they did not seem to receive much attention. Studies have confirmed that during the severe phase of the epidemic, COVID-19 elicits a strong psychological response among public health workers (13). In China, although they rarely directly work with clinical patients, most of them are involved in epidemic prevention and control at primary health organizations, the Centers for Disease Control (CDC), and hospital administrations. Over the past 3 years, personnel at these agencies have been primarily responsible for the epidemiology, viral etiology testing, data collection, and reporting of patients with confirmed and suspected COVID-19. The outbreak of the epidemic has brought a huge burden to their daily works, increased the detection rate of psychological problems, and reduced their professional identities and stabilities (14). Although the pandemic is much better, there are still many uncertainties and the long-term harm of the pandemic may persist, which is undoubtedly a challenge for public health and preventive medicine students who are going to engage in public health, and they may face high employment pressure and many psychological problems for a long time. Thai et al. (15) first reported that 80% of public health and preventive medicine students had a certain level of stress during COVID-19, but they did not identify the associated factors behind the stress, so there was no way to intervene. The purpose of this study was to understand the pressure of the COVID-19 pandemic on public health and preventive medicine students in terms of employment and career selection. Our hypothesis was that some of these students were hesitant to pursue a career in public health because the pandemic is not completely over. Understanding the stress level and related factors was an important prerequisite for finding ways to stabilize the public health workforce.

## 2. Materials and methods

### 2.1. Participants and procedures

The study was conducted through a cross-sectional online survey in March 2023 in Changsha, Hunan Province, China, which had seen multiple COVID-19 outbreaks in the past 3 years, involving every administrative district. We identified 2 universities, Central South University and Hunan Normal University, which were the only universities in Changsha to offer public health and preventive medicine majors. Both of which have independent schools or departments of public health, and both confer bachelor’s and master’s degrees. There were 620 students in the 2 universities and all students were invited to participate in the survey.

This study was approved by the Ethics Committee of Hunan Normal University School of Medicine (approval number: 2022-332;), and an informed consent was attached to the questionnaire to ensure that each student participated voluntarily. According to the lists provided by the 2 universities, we sent electronic questionnaires to each student by email and issued regular reminders. Students who did not reply more than three times were regarded as declined. All surveys were conducted anonymously, and no personal information would be seen in the background except IP address, and no personal information would be divulged. All items in the questionnaire were required. If any items were omitted, the questionnaire cannot be submitted. If a student was consistent in the choice of some variables (e.g., all 1), or had obvious logical errors, the questionnaire was considered invalid. The data in this study were all self-reported, which may introduce some bias and limit the ability to draw causal conclusions, but the setting of the census was still expected to identify some of the problems with these students.

### 2.2. Instruments

#### 2.2.1. Demographic characteristics and employment attitude

A self-made questionnaire was developed to collect participants’ demographic characteristics and employment attitudes. The former includes age, gender, grade, major, family residence, household income, intern experience, and COVID-19 vaccination. The latter includes whether they study their major voluntarily, their preferred employment intention, views on the impact of COVID-19 on employment, whether they want to change careers and the reasons for it, and whether they or others around them have been diagnosed or participated in the fight against COVID-19.

#### 2.2.2. Stress

The perceived stress scale 10 (PSS-10) was used to assess students’ perceived stress in the last month. The scale consists of 10 questions and is scored on a five-point scale from 0 (never) to 4 (often) (16). The highest score is 40, with higher scores indicating higher levels of stress, and scores of 13 and 26 were used as cut-offs for low, moderate, and high stress levels. The reliability and validity of the scale had been widely confirmed to be within the acceptable range, with an overall Cronbach’s alpha value of 0.82, the test-retest reliability coefficient of 0.69, and an internal consistency of 0.68–0.78. The scale was also found to be strongly correlated with PSS-4 (*R* = 0.95). In order to identify students’ stress levels after the pandemic, we highlighted in the questionnaire instructions: In the past month, how often have you experienced the following due to COVID-19?

#### 2.2.3. Anxiety

The general anxiety disorder 7 (GAD-7) was used to assess students’ anxiety symptoms after COVID-19. This is a validated screening tool for major anxiety disorders such as generalized anxiety disorder or panic disorder based on the seven-item symptoms (17). Students rated the frequency of these symptoms over the past 2 weeks. The severity of anxiety was classified according to the score as minimal or none (0–4), mild (5–9), moderate (10–14), and severe (≥15). The scale’s internal consistency coefficient was 0.92, the test-retest reliability was 0.83, and the correlation *R* with the Beck anxiety inventory (BAI) and the symptom check list-90 (SCL-90) were 0.72 and 0.74, respectively (18, 19).

#### 2.2.4. Loneliness

We used the University of California at Los Angeles loneliness scale 20 (UCLA-20) to assess students’ loneliness after the COVID-19 pandemic. The scale consisted of 20 items, and students were asked to choose 1 from 4 answers (1 = I have never felt this way, 4 = I have always felt this way). Nine items were scored in reverse order, and higher scores indicate higher loneliness. The scale divides loneliness into 5 levels: high loneliness (≥45), moderately high loneliness (39–44), moderate loneliness (34–38), moderately low loneliness (28–33), and low loneliness (0–27). The scale with an internal consistency of 0.89–0.94 and a test-retest reliability of 0.73 (20).

#### 2.2.5. Post-traumatic stress disorder

The PTSD checklist-civilian version (PCL-C) was used to assess for post-traumatic stress disorder (PTSD) after the COVID-19 pandemic (21). Students reported the degree of impact from the stressful life event during the past month on a scale from 1, indicating “not at all,” to 5, indicating “extremely.” This resulted in a total score that ranged from 17 to 85. Results were recorded dichotomously based on a cutoff score of 38.

### 2.3. Statistical analysis

Descriptive statistics (i.e., frequencies, percentages, means, SD) were calculated to describe students’ demographics, employment attitudes, and scores on psychometric scales. Two-sided *t*-tests or ANOVA tests were used to examine differences in PSS scores among multiple variables. Pearson correlations were calculated to examine associations between scores on psychological scales. A hierarchical multiple regression with 4 consecutive steps was used to detect factors associated with stress. Step 1 includes demographic variables. In step 2, we examined the association of employment attitudes with stress after controlling for demographic variables. In steps 3 and 4, we tested the association of anxiety and loneliness symptoms with stress after controlling for demographic variables and employment attitudes, respectively, PTSD was not included in the regression because it was homogeneous with stress. At each step, a backward procedure was used to remove relevant factors that did not significantly increase the prediction of stress. Before conducting the regression analysis, collinearity tests and Durbin–Watson (DW) tests were incorporated. All analyses were performed using SPSS 22.0 (SPSS/IBM, Armonk, NY, United States), and a *p*-value <0.05 was considered statistically significant.

## 3. Results

### 3.1. Sample characteristics

A total of 620 questionnaires were distributed and 539 were returned, with a recovery rate of 86.9%. Out of the returned questionnaires, 504 were valid, leading to an effective recovery rate of 93.5%. As shown in Table 1, the age of the students ranged from 18 to 26 years old (21.5 ± 2.6), 69.2% were female and 39.9% were only children. Students majoring in public health and preventive medicine were 38.9% and 61.1%, respectively. A total of 154 students (30.6%) had off-campus internships and all students (100%) had received COVID-19 vaccines. There were 103 (20.4%) students showing severe anxiety, 83 (16.5%) reported high levels of loneliness, 42 (8.3%) and 125 (24.8%) were detected with PTSD and high-stress levels.

**Table 1:** Demographic characteristics (*N* = 504).

Variable	Frequency	Prevalence (%)
*Age, years*		
≤20	220	43.7
21–24	225	44.6
≥25	59	11.7
*Gender*		
Male	155	30.8
Female	349	69.2
*Grade*		
1–2	94	18.7
3–4	232	46.0
≥5	178	35.3
*Major*		
Public health	196	38.9
Preventive medicine	308	61.1
*Family residence*		
Rural	237	47.0
Urban	267	53.0
*Only child*		
No	303	60.1
Yes	201	39.9
*Household income*/*month (¥)*		
<4,000	135	26.8
4,000–8,000	200	39.7
>8,000	169	33.5
*Intern experience*		
Without	350	69.4
Have	154	30.6
*Receive COVID-19 vaccine*		
No	0	0
Yes	504	100.0
*GAD score*		
<15	401	79.6
≥15	103	20.4
*UCLA score*		
<45	421	83.5
≥45	83	16.5
*PTSD score*		
<38	462	91.7
≥38	42	8.3
*PSS core*		
<26	379	75.2
≥26	125	24.8

### 3.2. Employment attitudes

A total of 84.7% of the students indicated that they voluntarily enrolled in this major. Among the employment intentions, most students chose to go to the hospital (37.3%), followed by CDC (23.0%), and only 0.8% were willing to go to primary health institutions. 18.5% hold that the epidemic had a negative impact on employment, and 12.5% had the idea of changing careers, mainly because they were worried about the increase in workload caused by the epidemic (8.1%). 96.6% of the students said that they or those around them had been diagnosed with COVID-19, and 28.8% participated in epidemic prevention and control (Table 2).

**Table 2:** Employment attitudes during COVID-19 pandemic (*N* = 504).

Variable	Frequency	Prevalence (%)
*Volunteer to study this major*		
No	77	15.3
Yes	427	84.7
*Preferred employment intention*		
Primary health institutions	4	0.8
Hospital	188	37.3
CDC^a^	116	23.0
Schools/research institutes	107	21.2
Pharmaceutical enterprises	14	2.8
Enter a higher school	60	11.9
Other	15	3.0
*Impact of COVID-19 on employment*		
Negative impact	93	18.5
No impact	63	12.5
Positive impact	348	69.0
*Idea of changing careers*		
Without	244	48.4
Have	63	12.5
Not sure	197	39.1
*Reasons for wanting to change careers*		
Worried about the workload of the corresponding majors under the epidemic	41	8.1
Worried about limited career prospects	19	3.8
The wishes of the parents	3	0.6
*Diagnosis of COVID-19 in oneself or someone close to you*		
Without	17	3.4
Have	487	96.6
*Participate in COVID-19 prevention and control work by oneself or someone close to you*		
Without	359	71.2
Have	145	28.8
*Forms of participation in the fight against COVID-19*		
Publicize knowledge of epidemic prevention and control offline and online	32	6.3
Participate in volunteer activities for epidemic prevention and control	293	58.1
Other	34	6.7

a Centers for Disease Control.

### 3.3. Comparison of different variables on PSS scores

The outcomes showed that the PSS scores of students had statistically significant differences in age, gender, grade, major, household monthly income, internship experience, the voluntary study of the major, the impact of the epidemic on employment, the idea of changing careers, and the participation in epidemic prevention and control (*p* < 0.05). In contrast, there were no statistically significant differences in family residence, only child, and diagnosis of COVID-19 (*p* > 0.05) (Table 3). Based on Pearson correlations, there were significant positive correlations among the GAD score, UCLA score, PTSD score, and PSS score (Table 4).

**Table 3:** Comparison of PSS score among variables during COVID-19 pandemic (*N* = 504).

Variable	Mean	SD	*t*/*F*	*p*
*Age, years*				
≤20	20.1	3.5	19.800	<0.001
21–24	24.7	4.4		
≥25	24.8	6.4		
*Gender*				
Male	26.3	7.7	4.658	<0.001
Female	23.3	3.8		
*Grade*				
1–2	20.2	2.9	38.812	<0.001
3–4	24.6	6.4		
≥5	25.9	4.0		
*Major*				
Public health	25.2	2.7	2.850	0.005
Preventive medicine	23.6	4.5		
*Family residence*				
Rural	23.8	4.4	−1.758	0.079
Urban	24.6	6.3		
*Only child*				
No	24.2	4.2	−0.283	0.778
Yes	24.3	7.1		
*Household income*/*month*				
<4,000	26.4	8.1	15.589	<0.001
4,000–8,000	23.5	3.3		
>8,000	23.3	4.6		
*Intern experience*				
Without	23.6	4.4	−3.475	0.001
Have	25.7	7.2		
*Volunteer to study this major*				
No	25.3	2.8	3.383	0.001
Yes	23.8	6.0		
*Impact of COVID-19 on employment*				
Negative impact	25.6	6.8	6.793	0.001
No impact	23.9	4.6		
Positive impact	22.5	3.4		
*Idea of changing careers*				
Without	23.4	6.5	3.321	0.037
Have	25.4	3.8		
Not sure	24.3	5.0		
*Diagnosis of COVID-19 in oneself or someone close to you*				
Without	25.3	9.6	0.334	0.743
Have	24.2	5.0		
*Participate in COVID-19 prevention and control work by oneself or someone close to you*				
Without	23.9	6.2	−2.928	0.004
Have	25.1	2.9		

PSS, perceived stress scale.

**Table 4:** Associations between scores of several psychological scales.

	GAD score	UCLA score	PTSD score	PS score
GAD score	1	0.350^**^	0.246^**^	0.637^**^
UCLA score	0.350^**^	1	0.117^**^	0.351^**^
PTSD score	0.246^**^	0.117^**^	1	0.548^**^
PSS score	0.637^**^	0.351^**^	0.548^**^	1

^**^p < 0.01.

### 3.4. Multivariate hierarchical regression analysis of student stress

The results of the collinearity test and DW test showed that the tolerance of all variables was greater than 0.1, and the variance inflation factor (VIF) was less than 10, indicating that there was no multicollinearity between variables. The value of DW was 1.6, meaning that the residuals were independent. As shown in Table 5, in step 1, the results showed that higher grade ( $B_{1}$ = 5.73, *p* < 0.001, $B_{2}=$ 7.74, *p* < 0.001), and internship experience (*B* = 1.14, *p* = 0.026) were significantly associated with higher stress levels while being female (*B* = −2.92, *p* < 0.001), majoring in preventive medicine (*B* = −1.85, *p* < 0.001), and higher monthly household income ( $B_{1}=-$ 2.12, *p* < 0.001, $B_{2}=-$ 2.09 *p* < 0.001) were negatively related to stress levels. In step 2, after controlling for demographic variables, those who believed that COVID-19 had a negative impact on employment (*B* = 3.94, *p* < 0.001) and participated in epidemic prevention and control (*B* = 1.29, *p* = 0.019) were associated with higher levels of stress. And those who hold that COVID-19 had a positive impact on employment (*B* = −2.98, *p* < 0.001) showed lower stress level associations. In step 3, after controlling for demographic variables and employment attitudes, higher GAD scores (*B* = 0.68, *p* < 0.001) showed a significant correlation with higher stress levels. In step 4, after controlling for all the above variables, the UCLA score detected a significant association with stress (*B* = 0.07, *p* = 0.002).

**Table 5:** Hierarchical regression analysis for factors associated with the PSS score in the post-pandemic period (*N* = 504).

Variable	Step 1				Step 2				Step 3				Step 4			
	Initial		Final^a^		Initial		Final^a^		Initial		Final^a^		Initial		Final^a^	
	*B*	*p*	*B*	*p*	*B*	*p*	*B*	*p*	*B*	*p*	*B*	*p*	*B*	*p*	*B*	*p*
Age, years	0.48	0.003	0.54	0.001	0.68	<0.001	0.58	<0.001	0.32	0.010	0.33	0.009	0.42	0.001	0.42	0.001
Gender	−2.68	<0.001	−2.92	<0.001	−2.81	<0.001	−2.89	<0.001	−1.79	<0.001	−1.84	<0.001	−1.64	<0.001	−1.64	<0.001
*Grade*																
3–4	5.52	<0.001	5.73	<0.001	5.31	<0.001	5.36	<0.001	3.53	<0.001	3.60	<0.001	3.59	<0.001	3.59	<0.001
≥5	7.46	<0.001	7.74	<0.001	7.64	<0.001	7.57	<0.001	4.29	<0.001	4.40	<0.001	4.57	<0.001	4.57	<0.001
Major	−1.77	<0.001	−1.85	<0.001	−1.26	0.009	−1.22	0.010	−0.32	0.424	—	—	—	—	—	—
Family residence	0.39	0.500	—	—	—	—	—	—	—	—	—	—	—	—	—	—
Only child	0.68	0.270	—	—	—	—	—	—	—	—	—	—	—	—	—	—
*Household income*/*month*																
4,000–8,000	−2.38	<0.001	−2.12	<0.001	−2.27	<0.001	−1.89	0.001	−0.46	0.326	—	—	—	—	—	—
>8,000	−2.51	<0.001	−2.09	<0.001	−2.38	<0.001	−1.98	0.001	−0.95	0.044	−0.65	0.090	—	—	—	—
Intern experience	1.09	0.035	1.14	0.026	1.24	0.021	1.05	0.043	0.98	0.021	0.99	0.020	1.16	0.006	1.16	0.006
Volunteer to study this major	—	—	—	—	−0.78	0.21	—	—	—	—	—	—	—	—	—	—
*Impact of COVID-19 on employment*																
Negative impact	—	—	—	—	3.81	<0.001	3.94	<0.001	2.57	<0.001	2.53	<0.001	2.56	<0.001	2.56	<0.001
Positive impact	—	—	—	—	−2.96	<0.001	−2.98	<0.001	−1.85	0.002	−1.83	0.002	−1.98	0.001	−1.98	0.001
*Idea of changing careers*																
Have	—	—	—	—	0.77	0.330	—	—	—	—	—	—	—	—	—	—
Not sure	—	—	—	—	1.02	0.051	—	—	—	—	—	—	—	—	—	—
*Diagnosis of COVID-19 in oneself or someone close to you*																
Have	—	—	—	—	1.26	0.34	—	—	—	—	—	—	—	—	—	—
*Participate in COVID-19 prevention and control work by oneself or someone close to you*																
Have	—	—	—	—	1.02	0.045	1.29	0.019	1.97	<0.001	1.90	<0.001	1.77	<0.001	1.77	<0.001
GAD score	—	—	—	—	—	—	—	—	0.68	<0.001	0.68	<0.001	0.64	<0.001	0.64	<0.001
UCLA score	—	—	—	—	—	—	—	—	—	—	—	—	0.07	0.002	0.07	0.002
*R*^2^	0.248		0.243		0.302		0.292		0.521		0.519		0.526		0.526	
Adj *R*^2^	0.232		0.226		0.281		0.276		0.509		0.507		0.516		0.516	

*B*: linear regression coefficient. The reference group of variables are as follows: gender: male; grade: 1–2; major: public health; family residence: rural; only child: no; household income/month: <4,000; intern experience: without; volunteer to study this major: no; impact of COVID-19 on employment: no impact; idea of changing careers: without; diagnosis of COVID-19 in oneself or someone close to you: without; participate in COVID-19 prevention and control work by oneself or someone close to you: without. GAD, general anxiety disorder; UCLA University of California at Los Angeles loneliness scale; PSS, perceived stress scale.

a Final models dropped nonsignificant variables using the backward procedure.

## 4. Discussion

This study focused on a detailed investigation of stress levels and related factors among students majoring in public health and preventive medicine in the post-pandemic period. We found that quite a few students experienced various degrees of psychological problems. 20.4% of the students reported anxiety symptoms, higher than the anxiety level of Chinese medical students (regardless of major) (22). Students’ anxieties were related to the impact of the outbreak on their studies, such as changes in curriculum and the increase of online courses (23), as well as concerns about future employments (24). 16.5% of students reported a high level of loneliness. Although loneliness has a certain relationship with individual personality traits, it is highly context-dependent, and the lockdown measures during the epidemic were a special situation that caused these students to reduce a lot of original social contacts. A research has confirmed that social isolation due to COVID-19 lockdowns resulted in a marked increase of loneliness among emerging adults aged 18–25 years old (25). Although only 8.3% of the students were detected with PTSD, the rate was higher than the 3.4% of the average college student in China in the post-COVID-19 era (9). This might be related to the large investment of public health personnel during the outbreak, and the higher attention and participation of public health and preventive medicine students compared with ordinary college students. The average PSS score of students in this study was 24.2, similar to the outcomes of a previous study from 31 provinces in China, which showed that the PSS score of medical students during the COVID-19 was 24.1, higher than the 22.6 of non-medical students (26). This is probably because that medicine is a highly practical discipline and medical students are under greater academic and employment pressures, especially for those with a wide range of responsibilities (27).

Different from other medical majors, students majoring in public health and preventive medicine have more opportunities to directly face the impact of the epidemic in theoretical learning and practice, including the accurate understanding of the harm of the novel coronavirus, the uncertainty of the safety of nucleic acid testing, and the high workload of epidemiological investigation. Some students were even directly involved in local epidemic prevention and control before graduation, thus showing more psychological problems and higher-stress levels. We found that anxiety, loneliness, and PTSD were positively associated with students’ stress levels, which is similar to the findings of Ye et al. (26). Stress is closely related to negative psychology such as anxiety and loneliness, which could lead adolescent students to avoid coping, thereby increasing the severity of psychological stress (27, 28). Thai et al. (15) confirmed that even though public health students indicated using positive and approaching coping strategies, they still showed a high prevalence of stress during the COVID-19 pandemic.

In the employment intentions of the students, 37.3% expressed their willingness to work in a hospital, which was in line with the overall positive attitude of all medical students towards the medical profession (29, 30). However, we noticed that only 0.8% of the students indicated that they would like to work in primary health care institutions after graduation, which seemed depressing because primary health care institutions, mainly community health service centers, played a very important role in epidemic prevention and control. In the past 3 years, the vast majority of COVID-19 vaccination and nucleic acid testing in China had been conducted by community health service centers, which were also responsible for epidemiological investigation and data collection of infected and potentially infected patients. In addition, 18.5% of the students believed that the epidemic had a negative impact on employment, higher than the 14.8% reported in domestic research which targeted all medical students (31). Our interpretation was that there were not only worries about not being able to find a job but also concerns about the content and workload of future work. Although the global epidemic has stabilized, the virus has not completely disappeared, and its future development is still full of uncertainty. In China, the efforts and sacrifices made by public health personnel in the process of fighting against the epidemic were no less than those of clinical medical staff, which will undoubtedly affect the employment attitude and behavior of these students. We also found that 12.5% of the students had the idea of changing careers and were mainly worried that the excessive workload during the epidemic might indirectly confirm our hypothesis.

This study found that older age, higher grades, and male students were significantly associated with higher stress levels. Age and grade increased synchronously. The closer to graduation, the more pressure on students would naturally increase. On the one hand, they have to complete the graduation examination from school, and on the other hand, they have to face the pressures of employment and career selection, both of which would be affected by the epidemic. Higher stress levels in male students might be attributable to social expectations that they take on more responsibilities and experience more economic stress (32), or boys usually suffer more negative life events and receive less social support (29).

According to our findings, students with internship and pandemic prevention experiences were associated with higher levels of stress. Previous studies also agreed that medical interns were more stressed than non-interns (33). Combined with the particularities of specialties, medical interns needed to avoid errors in their daily work, while the safety of throat swab collection and medical procedures has been uncertain during the outbreak period (26). The internships of public health and preventive medicine students mainly focused on community health service centers, CDCS, or hospitals, which were the main institutions to fight against COVID-19. When students participated in epidemic prevention and control in these departments, they would feel the difficulties and potential risks of work in advance. Coupled with the overwork caused by the lack of manpower during the epidemic, students’ concerns about future employment and their stress levels would be further increased.

There were 18.5% of the students believed the epidemic had a negative impact on employment and was significantly associated with higher stress levels. The COVID-19 epidemic undoubtedly provided more employment opportunities for these students, because it was an inevitable trend that countries needed to develop their public health systems in the post-pandemic era. However, in the situation of high morbidity and certain mortality, making everyone feel comfortable working in the industry is a challenge. A qualitative study revealed that overly positive attitudes towards the healthcare industry could be challenged by demanding and potentially risky working conditions close to reality (34). Although studies have confirmed that medical students’ attitudes to practice have not significantly changed as a result of the pandemic (29, 30), few studies had further compared differences among different medical students, whose willingness to practice varies according to the scope, content, and risk of their work. Whether COVID-19 leaves or permanently coexists with human beings, the necessity for the development of public health cannot be ignored, and the psychological stress of practitioners needs more attention. Therefore, relevant departments should provide psychological interventions to these students as early as possible based on the results of this study.

There were three shortcomings in this study. First, the design of the cross-sectional study made it impossible to establish a causal relationship. Second, sampling and response biases may persist because of low responses due to Internet connectivity and limitations in self-reported data. Third, the data were obtained at only 2 universities in one province, which has experienced several severe outbreaks. However, the severity of outbreaks varies from region to region, and the results reported by subjects from different regions might be biased, so conclusions beyond the scope of the investigation should be drawn with caution. Expanding the study area, increasing the sample size, and maximizing random sampling are the directions for improving these issues and our future research efforts.

## 5. Conclusion

In conclusion, students majoring in public health and preventive medicine in Changsha, China experienced higher stress levels in the post-pandemic period, which was influenced by age, grade, gender, internship experience, anti-epidemic experience, employment attitude, anxiety symptoms, and loneliness symptoms. Conducting fundamental courses on COVID-19, one-on-one psychological counseling, and inviting successful public health professionals to share their experiences were expected to alleviate the stress. Although the COVID-19 pandemic seemed to be coming to an end, the pandemic has been a wake-up call. Strengthening the attention and intervention of potential public health personnel, that is, students of public health and preventive medicine, is an important prerequisite to defend against any future major public health events.

## Data availability statement

The raw data supporting the conclusions of this article will be made available by the authors, without undue reservation.

## Ethics statement

The studies involving human participants were reviewed and approved by Biomedical Research Ethics Committee, Hunan Normal University, 2022(332). The patients/participants provided their written informed consent to participate in this study.

## Author contributions

XW: designed the study and wrote the initial draft. JZ and NY: analyzed the data and interpreted results. PH: contributed content to subsequent drafts. XW, JZ, NY, MZ, and PH critically reviewed and approved the final draft. All authors contributed to the article and approved the submitted version.

## Funding

This study was supported by the Scientific Research Fund of Hunan Provincial Education Department (no. 21B0079), Changsha Natural Science Foundation Project (no. kq2202253), the Key Project of Hunan Provincial Evaluation Committee of Social Science Achievement (no. XSP20ZDI013), Open Project of Hunan Normal University Medical College (KF2022008), and Rehabilitation Project of Hunan Disabled Persons’ Federation (2023XK0216).

## Conflict of interest

The authors declare that the research was conducted in the absence of any commercial or financial relationships that could be construed as a potential conflict of interest.

## Publisher’s note

All claims expressed in this article are solely those of the authors and do not necessarily represent those of their affiliated organizations, or those of the publisher, the editors and the reviewers. Any product that may be evaluated in this article, or claim that may be made by its manufacturer, is not guaranteed or endorsed by the publisher.
